# Regulate and be regulated: integration of defense and other signals by the AtMYB30 transcription factor

**DOI:** 10.3389/fpls.2013.00098

**Published:** 2013-04-11

**Authors:** Sylvain Raffaele, Susana Rivas

**Affiliations:** ^1^INRA, Laboratoire des Interactions Plantes-Microorganismes, UMR441Castanet-Tolosan, France; ^2^CNRS, Laboratoire des Interactions Plantes-Microorganismes, UMR2594Castanet-Tolosan, France

**Keywords:** Arabidopsis, AtMYB30, hypersensitive response, MYB transcription factor, plant defense, stress responses, transcriptional regulation

## Abstract

Transcriptional regulation in host cells plays a crucial role in the establishment of plant defense and associated cell death in response to pathogen attack. Here, we review our current knowledge of the transcriptional control of plant defenses with a focus on the MYB family of transcription factors (TFs). Within this family, the Arabidopsis MYB protein AtMYB30 is a key regulator of plant defenses and one of the best characterized MYB regulators directing defense-related transcriptional responses. The crucial role played by AtMYB30 in the regulation of plant disease resistance is underlined by the finding that AtMYB30 is targeted by the *Xanthomonas* type III effector XopD resulting in suppression of AtMYB30-mediated plant defenses. Moreover, the function of AtMYB30 is also tightly controlled by plant cells through protein-protein interactions and post-translational modifications (PTMs). AtMYB30 studies highlight the importance of cellular dynamics for defense-associated gene regulation in plants. Finally, we discuss how AtMYB30 and other MYB TFs mediate the interplay between disease resistance and other stress responses.

## Introduction

As sessile organisms, plants must face the diversity of pathogens that they encounter in their habitat. Unlike mammals, plants rely on cell autonomous innate immunity and on systemic signals originating from infection sites (Jones and Dangl, 2006). Plant immunity is activated by multiple transcriptional regulators that switch cell transcription programs from routine cellular requirements to defense. The arsenal of transcriptional regulators includes DNA-binding transcription factors (TFs) and proteins that regulate these TFs. Plant transcriptional regulators function cooperatively in complex networks to control the speed, intensity, localization, and duration of the immune response (Moore et al., 2011). The rapid and localized programmed death of infected cells is part of a typical plant immune response designated as the Hypersensitive Response (HR) (Mur et al., 2008; Coll et al., 2011). Processes related to the sessile lifestyle of plants have been associated with the expansion of TF families controlling plant-specific functions (Dias et al., 2003; Shiu et al., 2005; Feller et al., 2011). The MYB family of TFs underwent an extensive amplification approximately 500 million years ago due to recent whole-genome duplications and segmental tandem duplication events (Shiu et al., 2005). As a result, the plant MYB family typically comprises hundreds of members, classified based on the number of MYB repeats that they contain (Feller et al., 2011). MYB R2R3 proteins contain two MYB repeats and form the largest group of MYB TFs in plants. Members of the R2R3 MYB family regulate mostly plant-specific functions, including immunity against microbial pathogens (Stracke et al., 2001; Dubos et al., 2010).

In *Nicotiana tabacum*, the expression of the *Ntmyb1* gene is induced during the response to Tobacco Mosaic Virus (TMV) and *Pseudomonas syringae* pv. *syringae* avirulent bacteria. The Ntmyb1 protein binds to the promoter of the defense-related gene *PR-1a* suggesting a role in the regulation of immune responses (Yang and Klessig, 1996). In an independent study, Ntmyb1 was retrieved together with three other R2R3 MYBs as factors binding to the promoter of defense-related genes (Sugimoto et al., 2000). Transgenic *N. tabacum* plants overexpressing the rubber tree *HbMyb1* MYB gene exhibited suppressed HR resulting in enhanced resistance to the necrotrophic fungus *Botrytis cinerea* (Peng et al., 2011). Conversely, overexpression of the wheat *TaPIMP1* MYB gene caused stronger HR and enhanced resistance to the biotrophic bacterial pathogen *Ralstonia solanacearum* in tobacco and to the hemibiotrophic fungal pathogen *Bipolaris sorokiniana* in wheat (Liu et al., 2011; Zhang et al., 2012). In rice, the *OsJaMyb* R2R3 MYB gene is induced during infection by the blast fungus *Magnaporthe oryzae* and in mutants altered in cell death programs suggesting a role in defense responses (Lee et al., 2001). The *Arabidopsis thaliana* genome harbors 137 R2R3 MYB genes some of which have been shown to regulate immunity to microbial pathogens. The *BOTRYTIS-SUSCEPTIBLE1 BOS1/AtMYB108* gene was identified in a screen for mutants altered in their response to the *B. cinerea*. The *bos1* mutant exhibits enhanced susceptibility to *B. cinerea* and *Alternaria brassicicola* necrotrophic pathogens and reduced symptoms but unaltered resistance in response to biotrophic pathogens (Mengiste et al., 2003). Conversely, AtMYB46 negatively regulates resistance to *B. cinerea* likely *via* the regulation of a cell wall-bound peroxidase (Ramirez et al., 2011). Overexpression and silencing of AtMYB44 demonstrated that it positively regulates resistance to the virulent bacterium *P. syringae* pv. *tomato* (*Pst*) DC3000 but down regulates resistance to *A. brassicicola via* the WRKY70 TF (Shim et al., 2012; Zou et al., 2012). AtMYB96 was first reported as induced upon *Cauliflower Mosaic Virus* infection (Geri et al., 1999). Analysis of plants mis-expressing *AtMYB96* demonstrated that this TF positively controls resistance to *Pst* DC3000 in a salicylic acid-dependent manner (Seo and Park, 2010). Among the closest paralogs of AtMYB96 is AtMYB30, which was the first R2R3 MYB gene to be associated with the regulation of defense response in *Arabidopsis* and one of the best defense-related MYBs characterized to date. Although the mechanisms by which MYB TFs control defense responses are still enigmatic, recent advances in our understanding of AtMYB30 function summarized in this review shed new light on the regulation of plant immunity by this family of TFs.

The *MYB* oncogene homologue *AtMYB30* was first isolated in by differential screening of a cDNA library prepared from *Xanthomonas campestris* pv. *campestris (Xcc)*-inoculated *Arabidopsis* cells (Lacomme and Roby, 1999). Early, transient and specific activation of *AtMYB30*, prior to the onset of the hypersensitive cell death, was observed after treatment with different avirulent bacterial pathogens (Daniel et al., 1999). In addition, overexpression of *AtMYB30* in *Arabidopsis* and tobacco led to acceleration and intensification of the HR, enhanced accumulation of HR molecular markers and increased resistance in response to avirulent pathogens. Conversely, the antisense-mediated downregulation of *AtMYB30* led to a strong decrease or suppression of the HR (Vailleau et al., 2002). These data identify AtMYB30 as a positive regulator of the signaling pathway controlling the establishment of cell death responses to pathogen attack.

During the last few years, the study of AtMYB30 regulatory mechanisms has increased our knowledge about the mode of action of this TF. These studies have uncovered a tight control of the activity of AtMYB30 through protein-protein interactions and post-translational modifications (PTMs). Here, we summarize our current knowledge of the AtMYB30 interaction and regulatory network involved in the control of plant defense responses. Additional roles of AtMYB30 during the integration of other environmental cues are also discussed.

## AtMYB30 regulates genes of the VLCFA pathway

**Figure 1:**
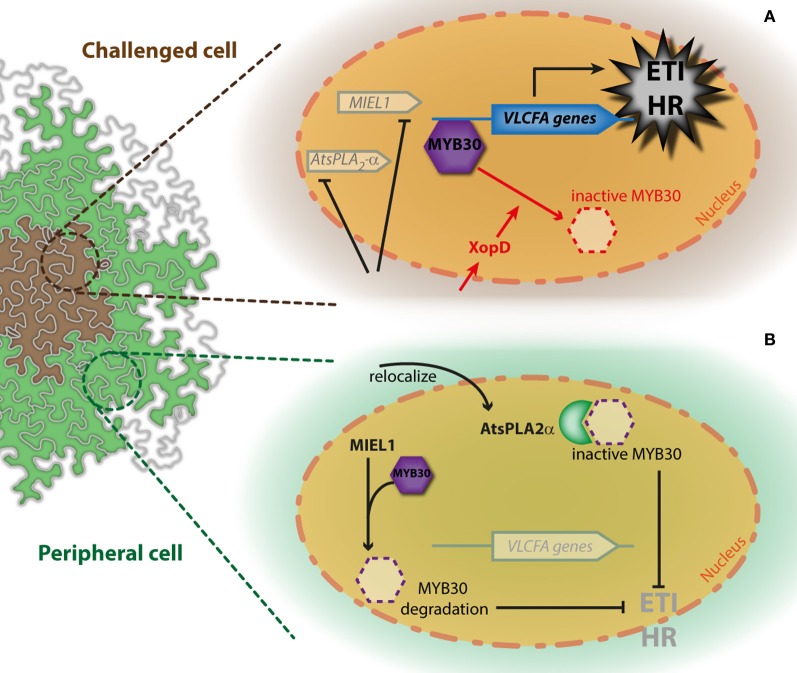
**Simplified model for the simultaneous regulation of AtMYB30-mediated HR cell death through interaction with *At*sPLA_2_−α and MIEL1.** The action of with *At*sPLA_2_−α and MIEL1 on AtMYB30-mediated HR development is presented in cells challenged with bacterial inoculation **(A)** and peripheral cells **(B)**. Activity of the bacterial XopD effector is shown in red. See the text for details.

**Figure 2:**
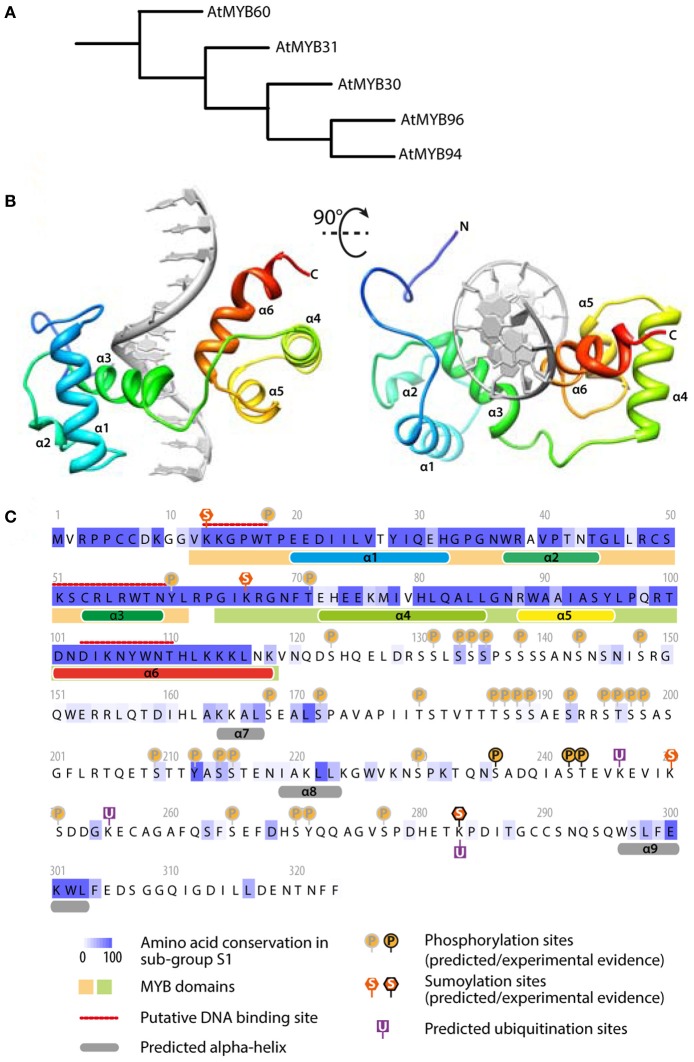
**AtMYB30 sequence analysis: relationship with other MYBs, protein motifs and predicted structure. (A)** Relationship between MYB TFs of the subgroup S1 (from Dubos et al., 2010). **(B)** Predicted structure of AtMYB30 DNA binding domain bound to DNA (gray). The model was predicted using the I-TASSER server and rendered with UCSF Chimera. **(C)** Sequence analysis of AtMYB30 protein. The conservation between members of subgroup S1 was inferred from a MUSCLE alignment and colored using JALVIEW. Alpha helices and DNA binding sites were predicted using the I-TASSER server. MYB domains were identified using INTERPROSCAN. Phosphorylation, sumoylation and ubiquitation sites were predicted using PhosphAt, Sumoplot and Ubpred respectively.

A transcriptomic analysis revealed that AtMYB30 putative target genes are involved in the lipid biosynthesis pathway that leads to the production of very long chain fatty acids (VLCFAs) (Raffaele et al., 2008). In good agreement, ectopic expression of *AtMYB30* activates genes encoding subunits of the acyl-coA elongase complex and alters the VLCFA content of *Arabidopsis* leaves. Furthermore, defense-related phenotypes of *AtMYB30* transgenic plants are dependent on the VLCFA biosynthesis pathway, supporting the view that AtMYB30 modulates cell death-related lipid signaling by enhancing the synthesis of VLCFAs or VLCFA derivatives (Raffaele et al., 2008) (Figure 1A). Downstream products of the VLCFA pathway include sphingolipids, wax and cutin. Wax synthesis was altered by *AtMYB30* over-expression but not by *AtMYB30* silencing, suggesting that sphingolipids could be cell death signals regulated by AtMYB30, and that activators of the wax synthesis pathway could compensate for the lack of AtMYB30 in silenced plants. Interestingly, Seo et al. reported that AtMYB96 activates genes of the wax biosynthesis pathway during drought stress (Seo et al., 2011). AtMYB30 and AtMYB96 belong to the sub-group S1 of *Arabidopsis* R2R3 MYB family (Figure 2A) (Dubos et al., 2010). Their N-terminal domain is predicted to mediate DNA-binding through a six alpha-helix domain typical of R2R3 MYBs (Figure 2B). AtMYB30 and AtMYB96 share extensive similarity in their N-terminal domain (Figure 2C), as expected considering the overlap in their respective lists of target genes. Besides short conserved motifs, the C-termini of sub-group S1 of MYB TFs are highly divergent. In AtMYB30, this C-terminal region harbors numerous putative regulatory sites, including phosphorylation, SUMOylation and ubiquitination sites (Figure 2C). As discussed below, modifications of this kind are critical for the regulation of AtMYB30 activity. It is therefore tempting to speculate that the differential activation of the N-termini of MYB TFs of the sub-group S1 may integrate signals arising from multiple stresses to regulate a partially common set of genes. Whether and how the interplay between AtMYB30 and AtMYB96 fine-tunes the activation of VLCFA-mediated responses remains to be investigated. Whether other MYBs of sub-group S1 are able to activate the VLCFA pathway is also unknown. Shared and specific functions of related MYB TFs may explain how expansion and diversification in this family contributed to the emergence of an integrated stress-response machinery in plants.

## Manipulation of AtMYB30 activity by bacteria

XopD from strain B100 of *Xanthomonas campestris* pv. *campestris* (*Xcc*B100) is a modular type III effector protein of 801 amino acids that presents a modular structure and contains different domains with varied biochemical activities (Canonne et al., 2012). XopD_Xcc B100_ is targeted to plant cell nuclei (Canonne et al., 2011; Kim et al., 2011) and may interact with chromatin and/or transcriptional units, leading to modulation of host transcription by affecting chromatin remodeling and/or TF activity (Kay and Bonas, 2009).

In agreement with the idea that plant TFs and/or regulators might be direct targets of XopD, XopD_Xcc B100_ was shown to target AtMYB30. XopD_Xcc B100_ expression leads to accumulation of AtMYB30 in XopD_Xcc B100_-containing nuclear foci but the physical interaction between XopD_Xcc B100_ and AtMYB30 is independent of AtMYB30 relocalization to nuclear foci, as both proteins are also able to interact in the nucleoplasm (Canonne et al., 2011). XopD_Xcc B100_ targeting of AtMYB30 leads to reduced activation of AtMYB30 VLCFA-related target genes and, therefore, to suppression of plant defense responses during *Xcc*B100 infection (Canonne et al., 2011) (Figure 1A). A helix-loop-helix (HLH) domain in XopD_Xcc B100_ is necessary and sufficient to mediate the interaction with AtMYB30 and repression of AtMYB30 transcriptional activation and plant resistance responses. Consistently, XopD from the 8004 strain of *Xcc* (XopD_Xcc 8004_), that does not present the HLH domain and localizes homogenously within plant cell nuclei, is not able to interact with AtMYB30 and has no effect on AtMYB30 transcriptional activation. Considering the modular structure of XopD, it is likely that this type III effector mediates multiple molecular (protein-DNA and protein-protein) associations and that, depending on the *Xanthomonas* strain/host plant interaction, XopD is able to target different host components to subvert plant defense. For example, XopD from *Xanthomonas euvesicatoria* (*Xcv*) desumoylates the SlERF4 TF to suppress ethylene responses and promote pathogen growth in tomato (Kim et al., 2013).

## Regulation of AtMYB30 activity through protein–protein interactions and post-translational modifications

Plant resistance to disease involves costly defense responses, closely connected to plant physiological and developmental processes. A typical example is the HR, which includes the development of a form of programmed cell death and needs to be tightly regulated to be not only efficient but also beneficial to the plant. As a result, mutants with constitutively active defense responses often present stunted growth and low fertility (Lorrain et al., 2003). Negative regulatory mechanisms of defense responses are used by the plant to attenuate the activation of defense-related functions and allow a balanced allocation of resources upon pathogen challenge (Journot-Catalino et al., 2006; Mukhtar et al., 2008). AtMYB30 being a positive regulator of plant defense and associated cell death responses, several mechanisms of negative regulation of its activity have been described.

The secretory phospholipase PLA_2_ protein *At*sPLA_2_−α controls auxin transport protein trafficking to the plasma membrane (Lee et al., 2010). *At*sPLA_2_−α localizes to Golgi-associated vesicles and is later secreted to the extracellular space (Froidure et al., 2010; Lee et al., 2010). Translocation of *At*sPLA_2_−α to the apoplast is enhanced after plant inoculation with avirulent bacteria, suggesting that *At*sPLA_2_−α may participate to the plant defense response in the apoplast (Jung et al., 2012). Interestingly, intracellular *At*sPLA_2_−α has also been involved in the non-enzymatic control of plant defense. Indeed, *At*sPLA_2_−α was identified as interacting with AtMYB30 in yeast (Froidure et al., 2010). In the presence of AtMYB30, *At*sPLA_2_−α was partially relocalized to the plant cell nucleus where these two proteins interact, leading to repression of the AtMYB30-mediated transcriptional activity. As a result, *Arabidopsis* HR and defense responses are suppressed, supporting the view that AtMYB30 transcriptional activity is required to mount an efficient defense response during bacterial infection (Raffaele et al., 2008; Froidure et al., 2010). Notably, *At*sPLA_2_−α nuclear targeting, interaction with AtMYB30, repression of AtMYB30 transcriptional activity and HR development appeared to be independent of *At*sPLA_2_−α enzymatic activity (Froidure et al., 2010). Therefore, *At*sPLA_2_−α was proposed to control AtMYB30-mediated response through interaction with AtMYB30, preventing the activation of its targets, rather than through a lipid signal produced by *At*sPLA_2_−α. Together, these data highlight the importance of dynamic nucleocytoplasmic protein trafficking for the regulation of the transcriptional activation related to defense (Rivas, 2012). Interestingly, *AtMYB30* expression is induced 4 h post-inoculation (hpi) in challenged cells but not in peripheral cells, whereas *At*s*PLA_2_-*α expression peaks 6 hpi in peripheral but not in challenged cells (Froidure et al., 2010). This suggests that *At*sPLA_2_−α may contribute to restrict the development of the HR to the inoculated zone, thereby preventing spreading of cell death throughout the leaf (Froidure et al., 2010) (Figure 1B).

An additional regulatory mechanism of AtMYB30 action was uncovered by the identification of the *Arabidopsis* RING-type E3-ubiquitin-ligase MIEL1 (AtMYB30-INTERACTING E3 LIGASE1) as an AtMYB30 interactor in yeast (Marino et al., 2013). MIEL1 is able to ubiquitinate AtMYB30 *in vitro*. In *Arabidopsis*, MIEL1 leads to AtMYB30 proteasomal degradation, downregulation of its transcriptional activity and suppression of plant defense responses (Marino et al., 2013). Indeed, Arabidopsis *miel1* mutant plants displayed enhanced HR and resistance after inoculation with avirulent bacteria. These phenotypes are AtMYB30-dependent and correlate with down-regulation of AtMYB30 target genes related to VLCFA metabolism (Marino et al., 2013). *MIEL1* expression is rapidly repressed in challenged cells, indicating that MIEL1 may negatively regulate plant HR and defense activation through degradation of the MYB30 protein in the absence of the pathogen (Marino et al., 2013; Figure 2B). Repression of *MIEL1* in challenged cells may release AtMYB30 negative regulation, increasing the intensity of the HR and limiting pathogen growth (Marino et al., 2013; Figure 2A). In addition, MIEL1-mediated degradation of AtMYB30 could contribute to the spatial restriction of the HR to inoculated cells since *MIEL1* expression remains constant in peripheral cells (Marino et al., 2013; Figure 2B). Work by Marino and co-workers shows the important role played by ubiquitination during the transcriptional control of the HR (Marino et al., 2012) and underlines the sophisticated fine-tuning of plant responses to pathogen attack.

PTM of AtMYB30 by SUMOylation has also been reported. AtMYB30 SUMOylation was first demonstrated after reconstitution of the SUMOylation cascade in *E. coli*, the lysine residue K283 being the major SUMOylation site (Okada et al., 2009; Figure 2C). SUMOylation of AtMYB30 K283 by the *Arabidopsis* SUMO E3 ligase SIZ1 was later confirmed in *Arabidopsis* protoplasts and demonstrated to be required for AtMYB30 function during abscisic acid (ABA) signaling (Zheng et al., 2012) (see below). However, whether and how SUMOylation of AtMYB30 affects AtMYB30-mediated defense responses remains to be determined.

Finally, the AtMYB30 C-terminal region is particularly rich in potential phosphorylation sites for several protein kinases (Figure 2C). The contribution of these phosphorylation sites to the plant defense response is still unknown but it is tempting to speculate that different combinations of PTMs on AtMYB30 may act as a molecular barcode, which would be important for the regulation of TFs controlling multiple processes (Benayoun and Veitia, 2009). Along these lines, the animal TFs p53 and c-Myc represent excellent paradigms that illustrate the sophistication of transcription regulation with different PTMs providing efficient regulation of TF stability, subcellular localization and activity (Meek and Anderson, 2009; Hammond-Martel et al., 2012; Luscher and Vervoorts, 2012).

## AtMYB30, a regulator of multiple signals beyond the response to microbes

In addition to its role as a positive regulator of defense responses, AtMYB30 is recruited for the regulation of other signaling processes. The phytohormone ABA plays an essential role during development and in response to abiotic and biotic stress. AtMYB30 SUMOylation by SIZ1 leads to AtMYB30 protein stabilization and affects AtMYB30-mediated transcriptional activation of several ABA-responsive genes (Zheng et al., 2012), underlining the importance of AtMYB30 SUMOylation during the regulation of ABA signaling. As a result, an *atmyb30* mutant is hypersensitive to ABA whereas *AtMYB30*-overexpressing plants are insensitive to ABA (Zheng et al., 2012). Conversely, *AtMYB96* overexpressing plants were found to be hypersensitive to ABA, but an *atmyb96* knockout mutant was still responsive to ABA, possibly due to functional redundancy within the MYB family (Seo et al., 2009). *AtMYB96* expression is induced by ABA and drought and the activation of some ABA-inducible genes is AtMYB96-dependent. Similar to AtMYB30, enhanced disease resistance conferred by AtMYB96 involves salicylic acid synthesis, suggesting that these two MYB TFs regulate cross-talks between hormone signaling pathways and contribute to the integration of signals originating from various stresses (Raffaele et al., 2006; Seo and Park, 2010).

An additional example of the diversity of AtMYB30 functions is the regulation of brassinosteroid (BR) signaling. BRs play important roles in several plant growth and developmental processes as well as during stress/disease resistance. BRs signal through the BES1 (*bri1*-ethylmethane sulphonate suppressor1)/BZR1 (brassinazole-resistant1) family of TFs. BR treatment induces *AtMYB30* gene expression in *Arabidopsis* seedlings and in *bes1-D* plants, that overexpress BES1, *AtMYB30* expression is upregulated, indicating that AtMYB30 may function in the BR signaling pathway (Li et al., 2009). Indeed, chromatin immunoprecipitation (ChIP) experiments showed that BES1 activates *AtMYB30* expression by directly binding to the *AtMYB30* promoter (Li et al., 2009). In agreement with this finding, *atmyb30* knockout mutant plants exhibit reduced BR-related gene expression and phenotypes, indicating that AtMYB30 promotes the expression of a subset of BR target genes (Li et al., 2009). Moreover, the promoters of *AtMYB30* and *BES1* common target genes harbor boxes bound by each TF. Finally, AtMYB30 and BES1 interact with each other. Together, this data shows that AtMYB30 functions to amplify BR signaling through cooperation with BES1 to promote BR target gene expression.

## Conclusions and perspectives

Cellular responses to environmental or physiological cues rely on transduction pathways that must discriminate between different signals and ensure a combinatorial regulation. Thus, combinations of different PTMs and protein-protein interactions provide different layers of information that may allow the integration of several transduction pathways and warrant highly specific cellular outputs. Accumulating evidence shows that the *Arabidopsis* MYB regulator AtMYB30 is a multi-regulated protein that is involved in the integration of various environmental stimuli, including attack by microbes, abiotic stress and hormone signaling, likely through the activation of shared and specific sets of target genes. How simultaneous and diverse stress signals are integrated into a unified cellular response is a major unknown in cell signaling. The acceleration of large data set acquisition and the development of systems biology approaches promise to offer new insights into the functioning of such complex regulatory networks. The wealth of knowledge gained in recent years on *Arabidopsis* R2R3 MYB TFs provides an excellent framework toward this end.

### Conflict of interest statement

The authors declare that the research was conducted in the absence of any commercial or financial relationships that could be construed as a potential conflict of interest.
